# Making UV light visible by exciting polarization-gate phototransistor to achieve energy transfer into GaN-based blue emission

**DOI:** 10.1038/s41377-026-02242-4

**Published:** 2026-03-10

**Authors:** Chunshuang Chu, Yao Jiang, Conglin He, Wenjie Li, Kangkai Tian, Yonghui Zhang, Xiaowei Sun, Zi-Hui Zhang

**Affiliations:** 1 School of Integrated Circuits, Guangdong University of Technology, Guangzhou, China; 2 State Key Laboratory of Reliability and Intelligence of Electrical Equipment, School of Electronics and Information Engineering, Hebei University of Technology, Beichen, Tianjin China; 3 Institute of Nanoscience and Applications, and Department of Electrical and Electronic Engineering, Southern University of Science and Technology, Shenzhen, China

**Keywords:** Inorganic LEDs, Photonic devices

## Abstract

In this work, we have made ultraviolet (UV) light visible by proposing and fabricating an integrated optoelectronic device. The demonstrated device consists of a GaN-based blue mini-light-emitting diode (mini-LED) and a phototransistor. The phototransistor is specially designed with an Al_0.20_Ga_0.80_N polarization gate. The background electrons can be depleted by the polarization gate to enable the normally-off state for the integrated optoelectronic device when there is no UV illumination. Our measured results show that when the polarization-gated phototransistor is switched off, the current for the integrated optoelectronic device is as low as 1.4 × 10^−4 ^mA even when the device is biased to 10 V. Upon the 12.7 mW UV excitation, the current for the integrated device can be increased to 44.4 mA at the bias of 10.0 V. This enables the GaN-based visible mini-LED to generate the optical power of 81.1 mW. The largest power ratio between the UV excitation light and the mini-LED light of 49.8 times can be achieved, indicating the advantage of monitoring weak UV light by using the proposed design.

## Abstract (web summary)

We integrate polarization-gated UV phototransistor and blue InGaN/GaN mini-LED. The photon-generated electrons in the UV phototransistor make the mini-LED generate blue emission. This makes UV light “visible” to naked eyes.

## Graphical abstract

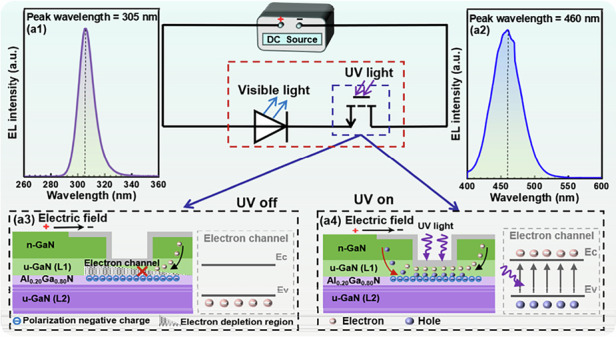

## Introduction

Ultraviolet (UV) light source has great application potentials in water sterilization, air purification, optical communication, biomedical sensing, etc.^1–3^. However, in spite of the tremendous advantages for UV light sources, long-time exposure under the high-energy UV radiation also poses significant health risks, including DNA damage, accelerated skin aging, photokeratitis- and cataract-related ocular disorders^2–4^. Hence, it is essentially important to detect the invisible UV light intensity so that the UV light source can maximize its function in protecting human beings.

AlGaN-based photodetectors (PDs) offer an effective solution by converting UV photon energy into photogenerated current, which can directly monitor the intensity of UV light. Potential structures include self-powered metal-semiconductor-metal (MSM) UV PDs, GaN p-i-n avalanche PDs, GaN-HEMT-based phototransistors and even in-situ monolithic integration of UV photodetector with UV light-emitting diode (LED)^5–9^. The multiple quantum wells (MQWs) can feature both functions of emission and absorption, which is regarded as a typical in-situ monolithic integration structure^10^. Nevertheless, considering the quantum-confined Stark effect (QCSE) and the Stokes shift, the MQWs shall be properly designed, such that the emissive MQWs shall produce a shorter wavelength so that the absorptive MQWs can effectively convert the photons to current^11^. To more easily detect UV light with naked eyes, visible LED modules with InGaN/GaN-based MQWs are commonly integrated into commercial UV photodetector chips during the packaging process. On the other hand, monolithically integrating a visible LED with UV LED by growing InGaN/GaN MQWs and AlGaN/AlGaN MQWs is also able to detect UV light^12^. It is noted that electrons have larger mobility than holes, which often leads to electron leakage for nitride-based LEDs^13,14^. As a result, when the integrated device is electrically biased, the leakage electrons from the AlGaN/AlGaN MQWs recombine with the holes in the InGaN/GaN MQWs, so that dual wavelengths are obtained. However, it is required that the UV emission shall be quickly probed, which requires that the visible InGaN/GaN LED shall be more efficiently controlled. It has been reported that light-emitting transistors (LETs) can respond much faster than LEDs^15,16^. InGaN/GaN-based gate-controlled LET has been firstly demonstrated by Schubert et al.^15^. The fabricated device adopts superlattice AlGaN/GaN structure on which the third gate terminal is designed. More holes can be generated when the gate bias is negative, and by doing so more holes can be injected into the MQWs, which helps to suppress the efficiency droop. The device on/off states can be more effectively controlled when the gate is deposited on the electrically resistive unintentionally n-type doped GaN (u-GaN) layer^17^. Such design can be further optimized to detect the UV emission by fabricating gate on the in-situ integrated AlGaN/GaN HEMT or MOSFET structure^18,19^. The gate shall be negatively biased so that the electron channel can be closed when the LED is turned off. The UV excitation source triggers the non-equilibrium carriers so that the electron channel in the HEMT or the MOSFET can be retrieved, which enables the current flow and the LED emission. However, to eliminate the usage of the third gate terminal, an NPN-structure below the InGaN/GaN MQWs is also designed^20^. The reversely biased PN-junction will block the electron injection so that the vertical LED is in the off-state. The UV excitation source enables the photon-generated carriers in the p-GaN layer, which favors the current flow through the NPN-junctions. The radiative recombination takes place in MQWs that indicates the detection of the UV light.

**Fig. 1:**
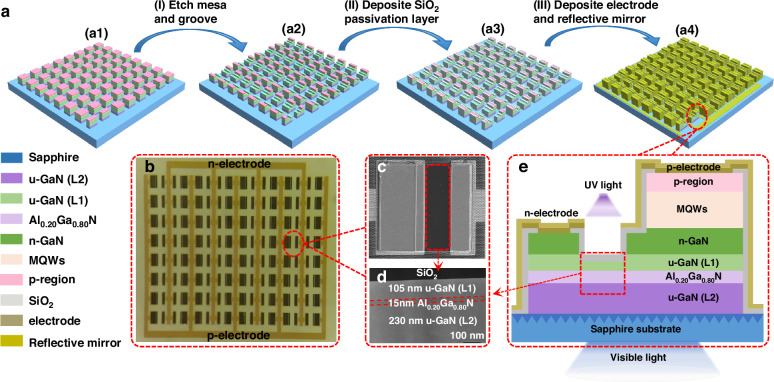
Device fabrication and structural characterization. **a** Schematic device fabrication processes, **b** top-view of optical microscopic image for the integrated optoelectronic device arrays, **c** top-view of SEM image for a single integrated optoelectronic device, **d** TEM image of the cross-sectional polarization-gated phototransistor structure, and **e** 2-D structural schematic for the single integrated optoelectronic device

At the current stage, considering the fabrication cost, most InGaN/GaN LEDs are grown on sapphire or Si substrates^21,22^. Hence, in this work, in order to quickly detect the UV light by using lateral mini-LED structure, we propose a monolithically integrated polarization-gated phototransistor (see Fig. 1). Different than others, the design of polarization-gate does not possess the third gate terminal. Instead, the proposed integrated device utilizes the polarization-induced negative charges at the u-GaN/AlGaN interface, which can form the electron depletion region and cuts off the electron channel in the u-GaN layer. Then, the current flow is prohibited when no UV excitation signal is applied. The photogenerated carriers excited by the UV light screen the polarization-induced electric field in the u-GaN layer so that the current flow can be enabled. This favors the light emission from the InGaN/GaN mini-LED for reflecting the ambient weak UV light.

## Results

The [0001]-oriented GaN-based integrated optoelectronic device is grown on a 4-inch nanopatterned sapphire substrates (NPSS) by using metal organic chemical vapor deposition (MOCVD) system. Device fabrications are depicted according to Fig. 1a. The microscopic top morphology for the fabricated arrays is shown in Fig. 1b. The bird’s-eye-view scanning electron microscopy (SEM) image for a single integrated optoelectronic chip is presented in Fig. 1c, in which the polarization-gated UV phototransistor is marked by the red dotted circle. To more clearly show each functional layer for the polarization-gated UV phototransistor, the transmission electron microscopy (TEM) image is shown in Fig. 1d, which is comprised of SiO_2_ insulation layer, u-GaN layer (L1) of 105 nm, Al_0.20_Ga_0.80_N layer of 15 nm and u-GaN layer (L2) of 230 nm. As schematically shown in the two-dimensional (2-D) structure diagram of Fig. 1e, GaN-based integrated optoelectronic device consists of a GaN-based visible mini-LED and a u-GaN (L1)/Al_0.20_Ga_0.80_N polarization-gated phototransistor. Because the SiO_2_ insulation layer is transparent to UV light, then the UV excitation light can be effectively received by the phototransistor. Detailed wafer epitaxial growth and device fabrication processes can be found in Materials and Methods.

**Fig. 2:**
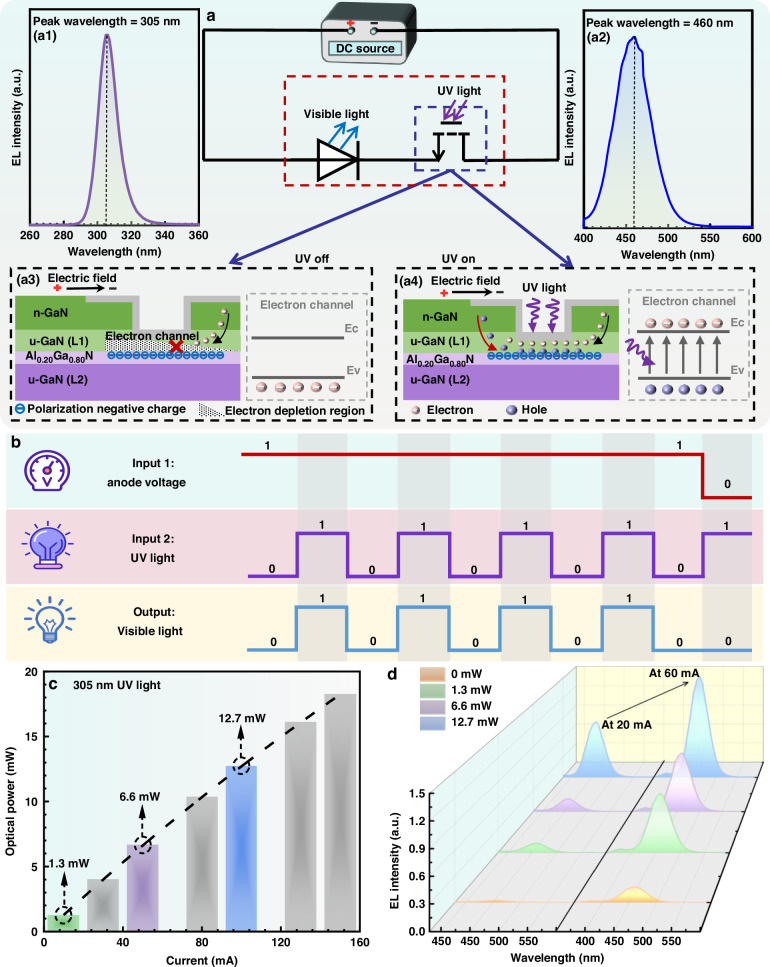
Working mechanism and optoelectronic characteristics of the integrated device. **a** Equivalent circuit diagram for the integrated optoelectronic device: EL spectra for (**a**1) UV excitation light source with the peak wavelength of 305 nm and (**a**2) mini-LED with the peak wavelength of 460 nm; Schematic phototransistor structure diagram, carrier generation process and carrier transport process for integrated optoelectronic device (**a**3) without UV excitation signal and (a4) with UV excitation signal. **b** Schematic visible light signal intensity in terms of UV light signal and the input anode voltage. **c** Optical power in terms of injection current for the UV excitation light source. **d** EL spectra for mini-LED in terms of the UV excitation light power biased at the injection current levels of 20 mA and 60 mA, respectively

The simplified equivalent circuit diagram and the working mechanism for the integrated optoelectronic device are illustrated in Fig. 2a. In the condition of no 305 nm UVB excitation [see Fig. 2a1], the phototransistor is in the off-state as shown in Fig. 2a3, so that the current fails to flow through the mini-LED. When the phototransistor receives the UV light with the peak wavelength of 305 nm [see Fig. 2a1] from a UVB LED chip, the phototransistor will be turned on and enables the mini-LED to generate the 460 nm emission as presented in Fig. 2a2, a4. Figure 2b presents the schematic logic sequence among the input bias, the external UV light signal and the visible light signal for our designed integrated device. Our device is a two-terminal design so that the on/off states for the visible LED is purely managed by the external UV signal without needing the third gate terminal. This makes the superiority for our design in programmable device control and electrical power saving. Figure 2c presents the optical power for the UVB LED chips in terms of the input current. We can obtain the optical powers of 1.3 mW, 6.6 mW and 12.7 mW at the current levels of 10 mA, 50 mA and 100 mA, respectively. The electroluminescence (EL) spectra in Fig. 2d tentatively show that the increased UV light power enables the enhanced blue emission intensity for the mini-LED. This proves the functionality for the integrated optoelectronic device in detecting UV light. Our fabricated integrated optoelectronic device is also able to detect weak deep ultraviolet (DUV) photons with peak wavelengths of 255 nm and 275 nm. Detailed investigations can be found in Supplementary Figs. S1, S2 in the Supplementary Material.

**Fig. 3:**
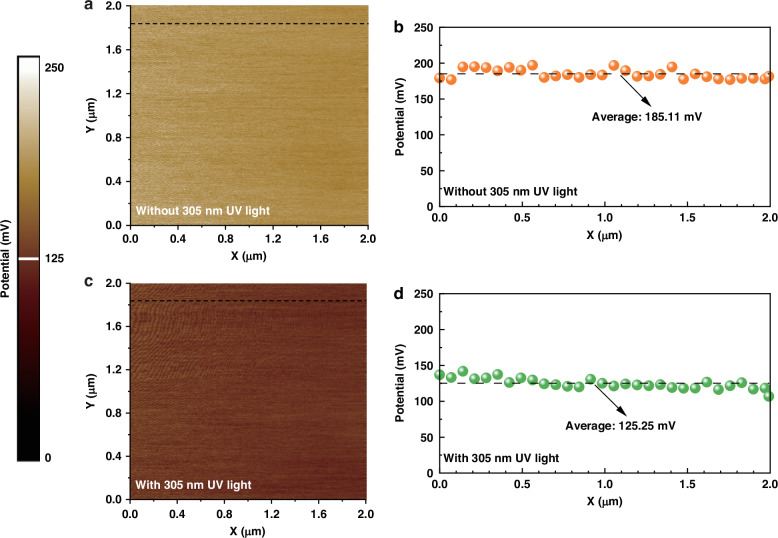
KPFM characterization of the polarization transistor region. **a** Surface potential difference mappings and **b** 1D surface potential difference profiles for the polarization-gated phototransistor in dark conditions. **c** Surface potential difference mappings and **d** 1D surface potential difference profiles for the polarization-gated phototransistor under 305 nm illumination

Figure 3a, b presents the Kelvin probe force microscopic (KPFM) potential mappings for the polarization transistor region in dark and UV illumination conditions, respectively. The probed area is 2 × 2 μm^2^. The UV light source for the KPFM characterization is 305 nm. Figure 3c, d shows the 1D surface potential profiles in the transistor in dark and UV illumination conditions. The surface potential differences between u-GaN(L1) layer and the Pt/Ir metal probe for the transistor are ~185.11 mV and ~125.25 mV in dark and UV illumination conditions, respectively. The reduced surface potential in the UV illumination condition well illustrates the generation for electrons.

**Fig. 4:**
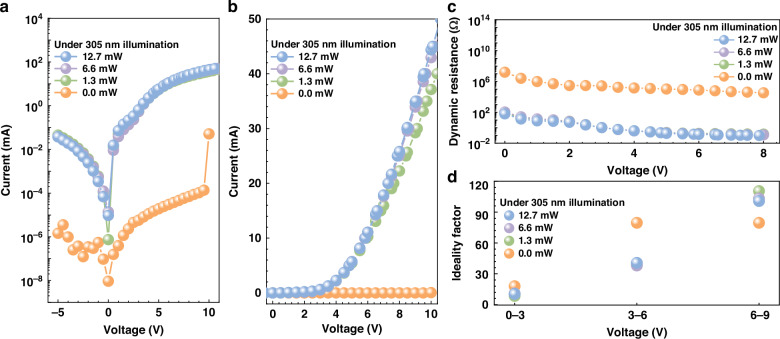
Electrical characterization of the visible mini-LED under UV excitation. Measured I-V characteristics in **a** semi-log scale and **b** linear scale, **c** dynamic resistances and **d** ideality factor for visible mini-LED in terms of the UV excitation light power

Figure 4a shows the semi-logarithmic current-voltage (I-V) characteristics for the integrated optoelectronic device in different 305 nm UV excitation conditions. Without 305 nm UV light illumination (i.e., 0.0 mW), the reverse leakage current is lower than 10^-6 ^mA in the tested bias range. The forward current is also smaller than 1.4 × 10^-4 ^mA when the bias is lower than 10 V. However, when the 305 nm UV light illuminates the device, thanks to the photo-generated current in the polarization-gated phototransistor, the forward current has been significantly increased. In the meanwhile, the UV light also gets the leakage current increased by ~4 orders of magnitude. Figure 4b shows the I-V characteristics in linear scale, which clearly shows that the turn-on voltage for the integrated optoelectronic device is ~3.2 V under the UV illumination. The increased UV light power also enables further increase to the current. Nevertheless, the increased UV excitation source power from 6.6 mW to 12.7 mW does not significantly enhance the current level. On the one hand, this is likely to be attributed to carrier-carrier scattering effect that reduces the electron mobility when more photogenerated carriers are obtained in the polarization-gated phototransistor^23,24^. On the other hand, the cathode produces electric field with small horizontal component that is perpendicular to [0001] orientation, and this correspondingly is not able to effectively drift electrons in the u-GaN(L1) layer. This ineffective electron injection also triggers current crowding effect [See Supplementary Fig. S3 in Supplementary Material].

Figure 4c then illustrates the dynamic electrical resistance in terms of the forward bias. It indicates that the device resistance is reduced by ~10^5^ times. Figure 4d demonstrates the ideality factors in three regimes. In the bias range between 0 V and 3 V, the average ideality factor is 9.9 for all three cases with UV excitation light. The average ideality factor is increased to 18.0 when no UV excitation light is applied, in which case the increased ideality factor well indicates the dominated defect-induced leakage current for the mini-LED when the photo-transistor is turned off^25–27^. In the bias range between 3 V and 6 V, when compared with 0.0 mW UV excitation light condition, the smaller average ideality factor further proves the carrier diffusion process for the mini-LED when the photo-transistor is turned on. When the bias is between 6 V and 9 V, the increased average ideality factors for the mini-LED become large when the UV excitation light is turn on. This well illustrates the occurrence for the current crowding effect [See Supplementary Fig. S3 in Supplementary Material], which normally takes place at very high current injection levels^28,29^. The mini-LED with the photo-transistor in the off-state has no chance of current crowding effect because of the very low carrier injection efficiency. The I-V characteristics, the dynamic resistance and the ideality factors for the fabricated integrated optoelectronic device under 255 nm and 275 nm DUV LED illuminations can be found in Supplementary Figs. S1, S2 in the Supplementary Material. The same conclusion has been obtained.

**Fig. 5:**
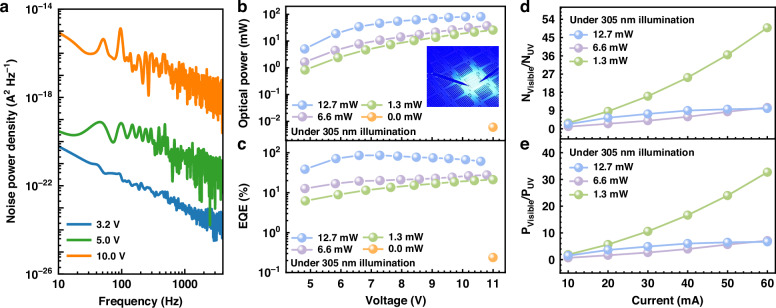
Comprehensive optoelectronic characterization of the integrated device. **a** Noise power density in term of the frequency at the forward biases of 3.2 V, 5.0 V and 10.0 V for our fabricated device. **b** Optical power and **c** EQE for visible mini-LED in terms of different applied biases when different UV excitation light powers are set, **d** N_visible_/N_UV_ and **e** P_visible_/P_UV_ in terms of the injection current for the integrated optoelectronic device

It is worth noting that, different than other photo-detectors that utilize the reversely biased junction to separate and transport the photon-generated carriers^30^, our device detects the UV signal in the forward-biased condition. We then measure the noise power density in terms of the frequency for our fabricated devices at the forward biases of 3.2 V, 5.0 V and 10. 0 V, which is presented in Fig. 5a. According to Fig. 4a, the fabricated device shows low dark current before the external UV light is turned on. The dependence of the noise power density on the applied bias agrees with Fig. 4a, such that the carrier generation-recombination (GR) process cannot be neglected when the forward bias is increased^30^. However, the measured noise power density is as low as 10^−20^ A^2^Hz^−1^ in low frequency regime, which indicates the possibility for our fabricated device in detecting weak UV emission.

Then, Fig. 5b, c demonstrates the optical power and the external quantum efficiency (EQE) produced by the mini-LED when the 305 nm UV excitation light powers are 0.0 mW, 1.3 mW, 6.6 mW and 12.7 mW, respectively. The optical power and the EQE produced by the mini-LED when the 255 nm and 275 nm DUV excitation light powers are applied can be found in Supplementary Figs. S4, S5 in the Supplementary Material. The optical power is collected from the sapphire side for our fabricated devices. According to Fig. 5b, when the drive voltage is 11.0 V and the UV excitation light power is 0.0 mW, the optical power for the visible mini-LED is 10^−2 ^mW. This indicates that the visible mini-LED is slightly turned on and accompanied by a weak emission. However, when the UV excitation source powers are increased to 1.3 mW, 6.6 mW and 12.7 mW, respectively, the optical powers for the mini-LED at the driving voltage about 10.2 V are increased to 21.6 mW, 32.0 mW and 81.1 mW, respectively. The optical power for the mini-LED also monotonically increases with the increasing UV excitation light power, which indicates that more photo-generated electrons are injected into the MQWs for the mini-LED. Figure 5c presents the EQE in terms of the applied bias. The conclusions agree well with Fig. 5b. In order to visually reveal the energy conversion efficiency between the UV light and the visible light for the integrated photonic device, Fig. 5d shows the ratio between the photon numbers for the 305 nm UV light and the 460 nm visible light (N_visible_/N_UV_) in terms of the injection current. We can find that the number of N_visible_/N_UV_ increases with the increased injection current. At the same time, a high N_visible_/N_UV_ of 49.8 can be achieved even when the external UV light power is low, e.g., 1.3 mW in the case for this work. Figure 5e shows the power-conversion efficiency (P_visible_/P_UV_) in terms of different external UV light power levels. Being consistent with Fig. 5d, a high power-conversion efficiency of 32.7 can be achieved even when the 1.3 mW external UV light power is low. This indicates that the fabricated devices are able to effectively probe weak UV light, i.e., making UV light “visible”. When the external UV light power is increased to 6.6 mW and 12.7 mW, the decreased N_visible_/N_UV_ probably originates from the electron leakage and the Auger recombination. This often causes the efficiency droop for blue and green InGaN/GaN LEDs biased at high injection current levels^31–33^.

**Fig. 6:**
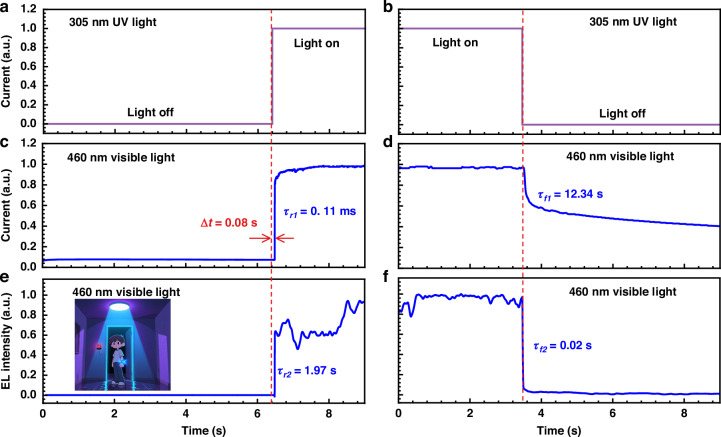
Switching characteristics of the integrated optoelectronic device under pulsed UV excitation. Dependence of the current and the blue emission on the pulsed 305 nm UV excitation light: **a** UV light is switched from off-state to on-state, **b** UV light is switched from on- state to off-state, **c** current is switched from off-state to on-state, **d** current is switched from on-state to off-state, **e** blue emission is switched from off-state to on-state, **f** blue emission is switched from on-state to off-state. The applied bias is 5 V. *τ*_*r1*_ and *τ*_*r2*_ represent the rise times for current and blue EL intensity, respectively. *τ*_*f1*_ and *τ*_*f2*_ represent the fall times for current and blue EL intensity, respectively

Figure 6 then demonstrates the dependence of the current and the blue EL intensity in terms of the pulsed UV light while the device is continuously biased at the bias of 5 V. Figure 6a, b demonstrates the pulsed signal transition from off-state to on-state and from on-state to off-state for the 305 nm external UV light, respectively. Figure 6c shows that there is a 0.08 s delay (Δ*t*) before the device current is triggered. The rise time for the current is 0.11 ms. Figure 6d indicates that the device current cannot be quickly turned off, and the fall time is 12.34 s. This time well be shortened when the device capacitance and deep-level defect density are both decreased. Figure 6e demonstrates the blue EL intensity quickly responds to Fig. 6c with the rise time of 1.97 s. When the UV light is turned off, the blue EL intensity drops with the fall time of 0.02 s according to Fig. 6f. However, the blue emission intensity will spend some time before completely decaying to 0, which is also addressed in Supplementary Fig. S6 in Supplementary Material.

According to Supplementary Fig. S7b and Supplementary Table S2 in the Supplementary Material, the minimum detected powers in our devices are 1.3 mW, 1.2 mW and 0.7 mW for the 305 nm, 275 nm and 255 nm wavelengths, respectively, which correspond to 130 mW·cm^−^^2^, 120 mW·cm^−^^2^ and 70 mW·cm^−^^2^ for the 305 nm, 275 nm and 255 nm wavelengths, respectively. Hence, the exposure durations per day are 0.385 s, 0.026 s and 0.083 s before the eyes can be injured, respectively. However, human beings can have their eyes open for 5 s on average in clean air. The 0.08 s response time is also sufficient for our eyes to be alerted by the blue emission when 305 nm UV light is exposed. Therefore, our devices are supposed to be able to alert human beings of UV emission. The blue emission that acts as visible light communication is likely to be observed by people in dark condition.

**Fig. 7:**
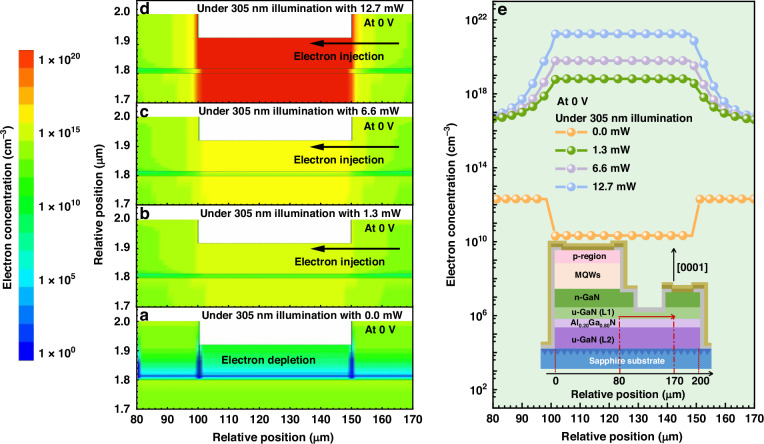
Simulated electron concentration profiles of the phototransistor at 0 V. **a**–**d** Calculated 2-D electron concentration distribution profiles and **e** 1-D concentration distribution profiles for the phototransistor in the integrated optoelectronic device. The data are calculated at the bias of 0 V

To better explain the impact of the UV excitation light on the carrier transport in the polarization-gated phototransistor and the mini-LED, we have conducted numerical simulations. Important simulation parameters can be found in the section of Simulations. We then present the two-dimensional (2-D) electron concentration profiles for the phototransistor without/with 305 nm UV light illumination in Fig. 7a–d, respectively. The 305 nm UV excitation source powers are set to 0.0 mW, 1.3 mW, 6.6 mW, and 12.7 mW, respectively with the UV light source of 305 nm peak emission wavelength. Figure 7a shows that the background electron concentration in the u-GaN (L1) layer for the polarization-gated phototransistor is depleted. This well interprets the very low current level in Fig. 4a. However, as shown in Fig. 7b–d, the electron concentration becomes significant in the u-GaN (L1)/Al_0.20_Ga_0.80_N/u-GaN (L2) structure for the phototransistor with the increasing 305 nm UV excitation light power. To even better present the electron concentration in different conditions, one-dimensional (1-D) electron concentration profiles are depicted in Fig. 7e. The electron concentrations in the u-GaN (L1) region are 2.1 × 10^10 ^cm^−3^, 6.5 × 10^18 ^cm^−3^, 6.2 × 10^19 ^cm^−3^ and 1.8 × 10^21 ^cm^−3^ when the UV excitation light powers are set to 0.0 mW, 1.3 mW, 6.6 mW, and 12.7 mW, respectively. Supplementary Fig. S8 in the Supplementary Material also depicts the electron concentration profiles at the applied bias of 3.2 V. It shows that, without the 305 nm UV illumination, the electrons concentration in the polarization-gated phototransistor is as low as 1.9 × 10^7 ^cm^−3^. This ensures the off-sate for the fabricated device, which also agrees with Fig. 4a, b.

**Fig. 8:**
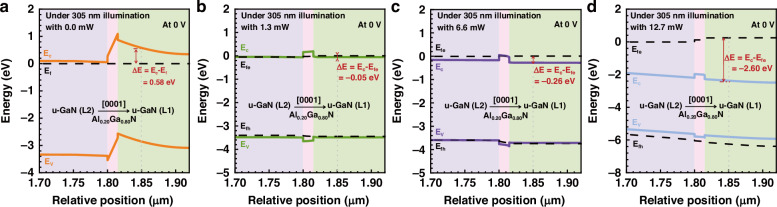
Simulated vertical energy band profiles of the phototransistor at 0 V. **a**–**d** Calculated energy band profiles of UV light detection region in the polarization-gated phototransistor for the integrated optoelectronic device at 0 V, respectively

To even better address the observations in Fig. 7, we then present the energy bands in the u-GaN (L1)/Al_0.20_Ga_0.80_N/u-GaN (L2) structure at the bias of 0 V in different UV illumination conditions in Fig. 8a–d, respectively. The strong electron depletion effect in u-GaN (L1) layer because of the Al_0.20_Ga_0.80_N polarization gate can be reflected by the energy difference between the conduction band and the Fermi-level (i.e., ΔE = E_c_-E_f_), which is 0.58 eV according to Fig. 8a. When the device is under 305 nm UV illumination with 1.3 mW, the electron depletion effect in the u-GaN (L1) layer has been significantly suppressed, such that ΔE is −0.05 eV [see Fig. 8b]. According to Fig. 8c, d, this number is further reduced to −0.26 eV and −2.60 eV when the device is under 6.6 mW and 12.7 mW 305 nm UV illumination conditions, respectively. Very strong electron concentration can be obtained when the Fermi-level for electrons is much higher than the conduction band, i.e., Fig. 8d. The energy band profiles are consistent with the electron concentration profiles Fig. 8e. The energy band profiles of UV light detection region in the polarization-gated phototransistor for the integrated optoelectronic device at 3.2 V can be found in Supplementary Fig. S9a–d in Supplementary Material.

## Discussion

In summary, in this work, we have proposed and fabricated GaN-based integrated optoelectronic device that consists of the phototransistor and the mini-LED. Our results show that the polarization-gated phototransistor is able to block the current flow by taking advantage of the polarization-induced negative charges at the GaN/AlGaN interface. Thanks to the electrical conductivity modulation effect by the photo-generated carriers, the phototransistor is able to support the current flow when the UV excitation source is applied, which also favors the mini-LED to generate blue emission. Our measured results show that, without the UV excitation source, the integrated optoelectronic device shows the very low current smaller than that of 1.4 × 10^−4 ^mA and the untestable blue emission from the mini-LED. Once upon being illuminated by the 12.7 mW 305 nm commercial UV LED, the current level and the optical power for the visible mini-LED are 44.4 mA and 81.1 mW, respectively. The visually visible blue emission proves the effectiveness for the demonstrated GaN-based integrated optoelectronic devices in UV light alert. Is it also worth emphasizing that our device is a two-terminal device with the polarization gate integrated into the device, which avoids any post-fabrication for gate metal deposition. Our device also reduces the control complexity when our device is transferred to flexible substrate for making wearable optoelectronics. Hence, we strongly believe that the proposed structure in this work helps the community make weak UV light visible by naked eyes. We also believe that the demonstrated approach also paves the way for making UV light and visible light communications.

## Materials and methods

### Epitaxy

The epitaxial growth for the GaN-based integrated optoelectronic device initiates from the 4-inch nanopatterned sapphire substrate. The epitaxial layers include an unintentionally n-type doped u-GaN (L2) buffer layer of ~1.8 μm, which is then followed by 200 nm/15 nm u-GaN (L1)/Al_0.20_Ga_0.80_N structure serving as the polarization-gated phototransistor. Then, a 1070 nm thick n-GaN layer is grown as the electron injection layer with the Si-doping concentration of 5 × 10^19 ^cm^−3^. Subsequently, five periods of 3 nm In_0.21_Ga_0.79_N/10 nm GaN multiple quantum wells are grown. Then, a 50 nm thick Al-gradient Al_0.15→0.0_Ga_0.85→1.0_N p-type layer and a 150 nm p-GaN layer are grown. The Mg-doping concentration for both layers is ~3 × 10^18 ^cm^−3^. Supplementary Fig. S10 in the Supplementary Material depicts the TEM image of the cross-sectional epitaxial layer for GaN-based integrated optoelectronic device.

### Fabrication

The devices are fabricated by following multi-step inductively coupled plasma (ICP) etching processes. We firstly conduct deep dry etching to form 200 μm × 200 μm mesas with the mesa depth of 1980 nm [see Fig. 1a1]. The deep etching is targeted to electrically isolate the individual integrated optoelectronic chip. The second dry etching with depth of 610 nm to expose the n-GaN electron injection layer and form visible mini-LED mesa of 80 μm × 200 μm. Then, we fabricate the polarization-gated phototransistor by selectively etching the n-GaN/u-GaN (L1) layers with depth of 830 nm to form 50 μm × 200 μm grooves [see Fig. 1a2]. The groove depth is 95 nm which is sufficient to expose the u-GaN (L1) layer. To alleviate the sidewall damages caused by mesa dry etching process, the epitaxial wafer is immersed in 20% KOH solution for 15 min before depositing a 300 nm SiO₂ insulating layer according to Fig. 1a3^34–37^. We use atomic layer deposition (ALD) to grown 20 nm SiO_2_ layer and the rest 280 nm SiO_2_ layer is grown by using plasma-enhanced chemical vapor deposition (PECVD). To decrease the optical absorption to the UV light by the SiO_2_ layer, rapid thermal annealing (RTA) for 5 min at the temperature of 650 °C in an N₂ ambient is conducted^38^. Ohmic contacts are fabricated by utilizing electron-beam evaporation system. The n-type Ti/Al/Ti/Au (20/30/60/100 nm) and the p-type Ni/Au (10/10 nm) metal stacks are deposited on the n-GaN layer and the p-GaN layer, respectively [see Fig. 1a4]. The post-deposition thermal annealing is performed at the temperature of 650 °C for 1 min in N₂ and at the temperature of 450 °C for 3 min in O₂ to reduce n- and p-type contact resistance, respectively. Finally, Ti/Al/Ti/Au (50/800/20/100 nm) reflective mirror structure is deposited, which interconnects the 8 × 8 single device into integrated optoelectronic device array [see Fig. 1b]. The reflective mirrors prevent the external UV light from being absorbed by other regions except the phototransistor.

### Characterizations

Structural properties of the samples are studied using JEM-2100F high-resolution field-emission transmission electron microscope and GAIA3 scanning electron microscopy-focused ion beam (SEM-FIB) dual-beam instrument. The EL spectra are collected by a calibrated integrating sphere with optical fiber and tested by high-resolution spectrometers, for which the model information is ATA-1000. The I-V characteristics are measured by using a Keithley 2400 voltage source meter. The Kelvin probe force microscopic system that measures the surface potential is Bruker Dimension FastScan. The noise power density is characterized by utilizing Keithley 2636B system. The response times for current and the blue emission are measures by using Keithley 2636B system and OHSP-350MUV wavelength-adjustable spectrum detector, respectively.

### Simulations

Numerical simulations are conducted by using Nuwa TCAD software. The absorption coefficients of AlGaN and GaN materials at different wavelengths can be found in ref. ^39^. The Auger recombination coefficient and Shockley–Read–Hall (SRH) recombination lifetime are set to 1.0 × 10^−30^ cm^6^·s^−1^ and 100 ns^40^, respectively. The energy band offset ratio for MQWs is 70:30^41^. The polarization effect is considered at each lattice-mismatched heterojunction, for which the polarization level is set to 40%^42^.

## Supplementary information

Supplementary Information

UV light detection

## Data Availability

The data that support the findings of this study are available from the corresponding authors upon request.
