# Supplementary material for: Making UV light visible by exciting polarization-gate phototransistor to achieve energy transfer into GaN-based blue emission
Source: Light Sci Appl. 2026 Mar 10;15:162. doi: 10.1038/s41377-026-02242-4 (PMC12976152; doi:10.1038/s41377-026-02242-4)
Supplement: Supplementary file 1 — Supplementary Information [file 41377_2026_2242_MOESM1_ESM.docx]

Supplementary Information for

Making UV light visible by exciting polarization-gate phototransistor to achieve energy transfer into GaN-based blue emission

Chunshuang Chu^1, †^, Yao Jiang^1, †^, Conglin He^1^, Wenjie Li^2^, Kangkai Tian^1^, Yonghui Zhang^2^, Xiaowei Sun^3^, Zi-Hui Zhang^1, 2, *^

^1^School of Integrated Circuits, Guangdong University of Technology, Guangzhou 510006, China

^2^State Key Laboratory of Reliability and Intelligence of Electrical Equipment, School of Electronics and Information Engineering, Hebei University of Technology, Beichen, Tianjin 300401, China

^3^Institute of Nanoscience and Applications, and Department of Electrical and Electronic Engineering, Southern University of Science and Technology, Shenzhen, 518055, China

^†^These authors contributed equally to this work.

^*^Corresponding author: [zh.zhang@hebut.edu.cn](mailto:zh.zhang@hebut.edu.cn)

Figures S1(a) and S2(a) show the semi-logarithmic current-voltage (I-V) characteristics for the integrated optoelectronic device in 275 nm and 255 nm DUV excitation conditions. When the 275 nm and 255 nm DUV light illuminates the device, the forward currents have been increased, respectively. The DUV light also gets the leakage current increased by ~4 orders of magnitude. Figures S1(b) and S2(b) show the I-V characteristics in linear scale, which clearly shows that the turn-on voltage for the integrated optoelectronic device is ~3.2 V under the DUV illumination. Figures S1(c) and S2(c) illustrate the dynamic electrical resistance in terms of the forward bias. It indicates that the device resistance is reduced by ~ 10^5^ times. Figures S1(d) and S2(d) demonstrate the ideality factors in three regimes. In the bias range between 0 V and 3 V, the average ideality factors are 10.2 and 9.4 for all the three cases with 275 nm and 255 nm DUV excitation light, respectively. The average ideality factor is increased to 18.0 when no DUV excitation light is applied. The same conclusion has been obtained for the fabricated integrated optoelectronic device under 275 nm and 255 nm DUV LED illuminations compared with that under 305 nm UV LED illumination.

**
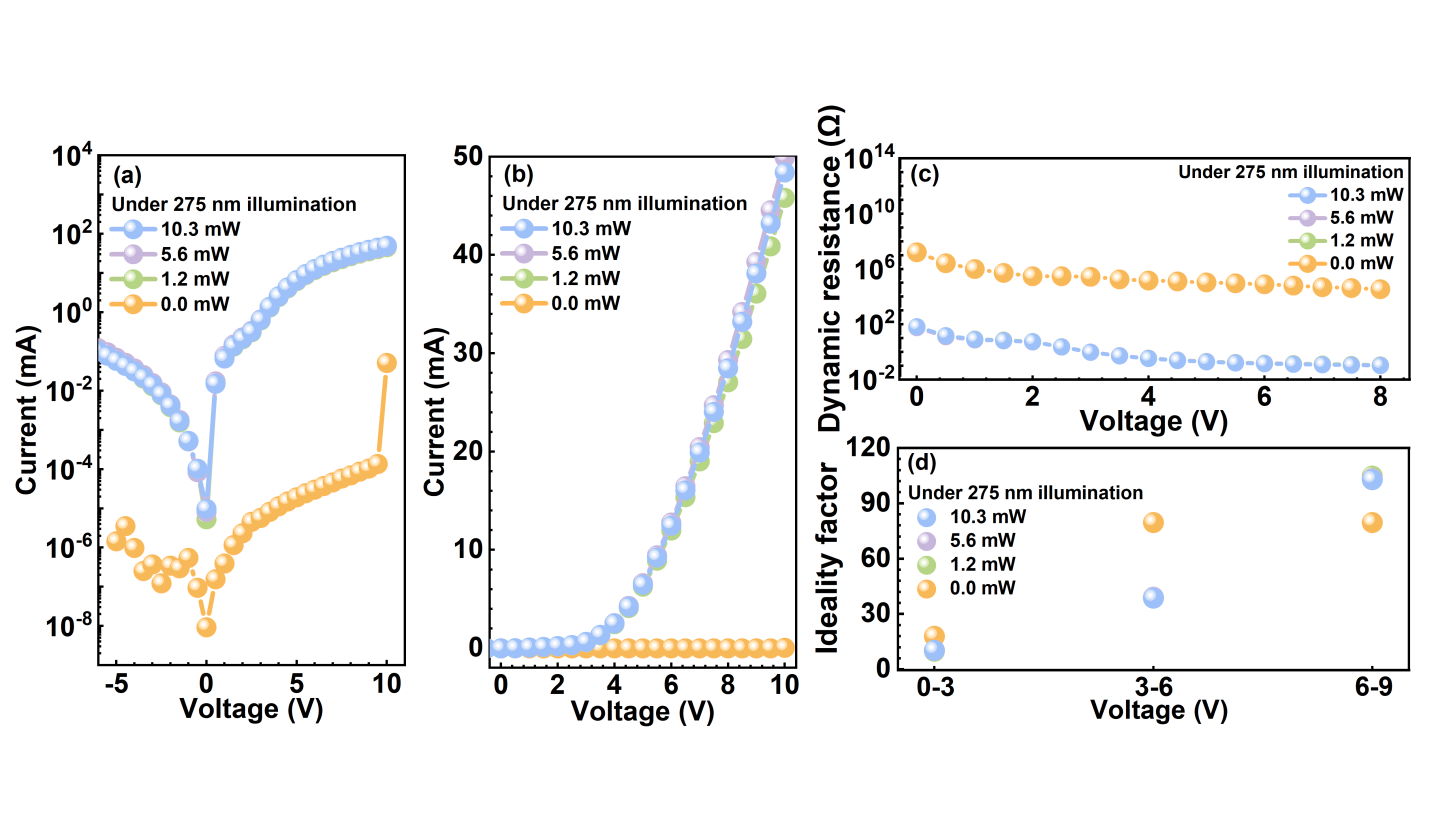
**

**Figure S1.** Measured I-V characteristics in (a) semi-log scale and (b) linear scale, (c) dynamic resistances and (d) ideality factor for visible mini-LED in terms of the 275 nm DUV excitation light power.

**
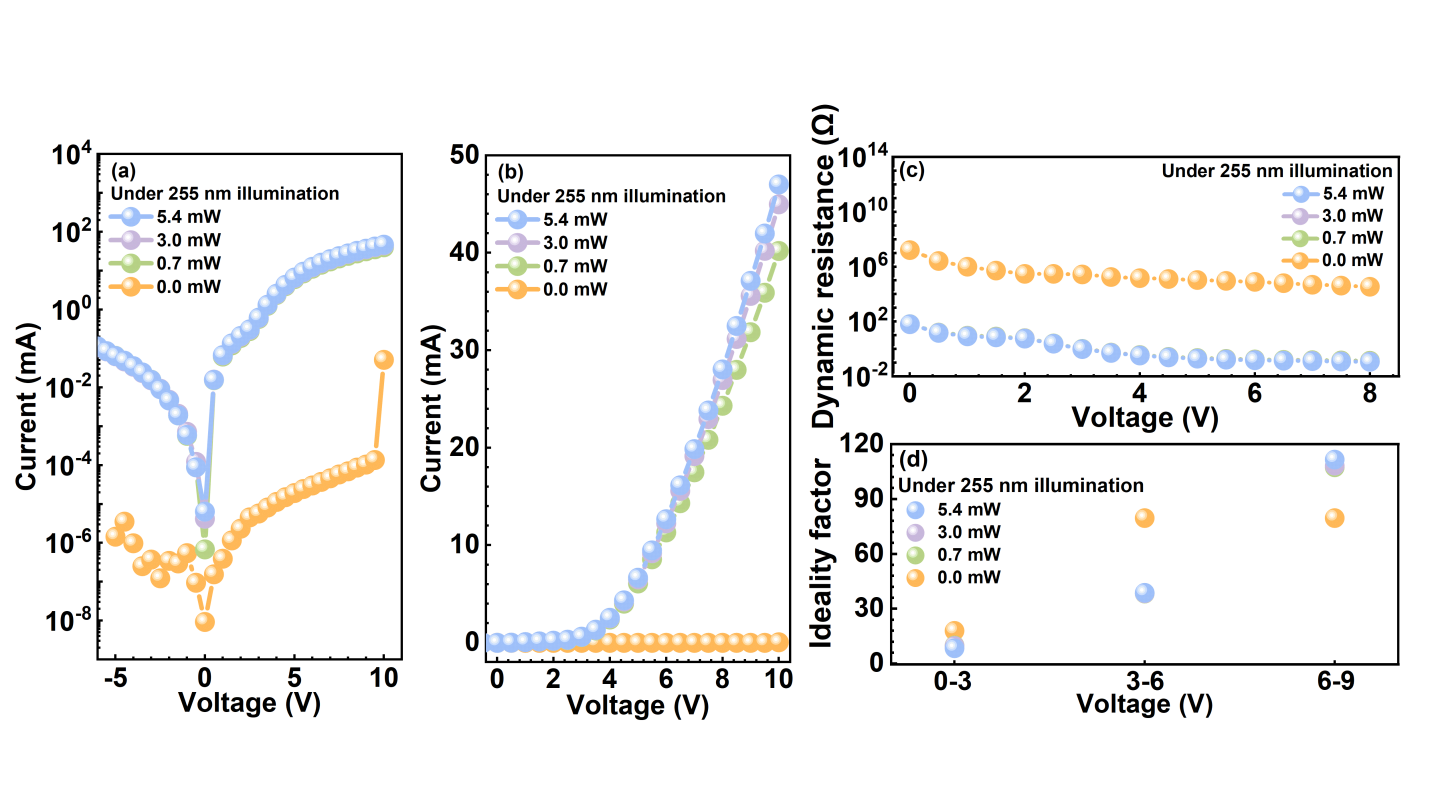
**

**Figure S2.** Measured I-V characteristics in (a) semi-log scale and (b) linear scale, (c) dynamic resistances and (d) ideality factor for visible mini-LED in terms of the 255 nm DUV excitation light power.

Figure S3 presents the 1-D and 2-D electron concentration profiles under the 305 nm UV illumination when an 8.0 V forward bias is applied. The average electron concentrations in the u-GaN (L1) layer region between 100 μm to 150 μm are 6.5 × 10^18^ cm^-3^, 6.2 × 10^19^ cm^-3^ and 1.8 × 10^21^ cm^-3^ when the UV excitation light powers are set to 1.3 mW, 6.6 mW, and 12.7 mW, respectively. This is because the electron depletion effect in the u-GaN (L1) layer has been significantly suppressed with the increased UV excitation light power. Hence, the local electron concentration increases with the increased UV light intensity. However, for the areas that are not directly exposed to UV light [e.g., the u-GaN (L1) layer region between 0 μm and 100 μm], we can see that the average electron concentration has slight variations with different UV excitation light powers, such that the electron concentrations are 2.5 × 10^8^ cm^-3^, 3.1 × 10^8^ cm^-3^ and 4.2 × 10^8^ cm^-3^, under the UV excitation powers of 1.3 mW, 6.6 mW, and 12.7 mW, respectively. This limits the electron injection efficiency and interprets the fact that the there is no significant increase of current when increasing the UV excitation light power.

It is noted that the polarization gate is enabled by both the u-GaN (L1) and the Al_0.20_Ga_0.80_N, neither of which are intentionally n-type doped. Hence, the electrical conductivity in the u-GaN(L1) layer is strongly subject to the nonequilibrium photon-generated rated electrons. However, the cathode for the fabricated device produces vertical electric field with small lateral contribution, which cannot promote the electron drift process and causes current crowding effect. This interprets the slight electron concentration variations in the u-GaN (L1) layer region between 0 μm and 100 μm with different UV excitation light powers.

We believe that when the cathode is also deposited on the sidewall, the lateral electrical field magnitude gets increased. This more effectively transport electrons so that the current can be remarkably increased with the enhanced UV excitation light source power.

**
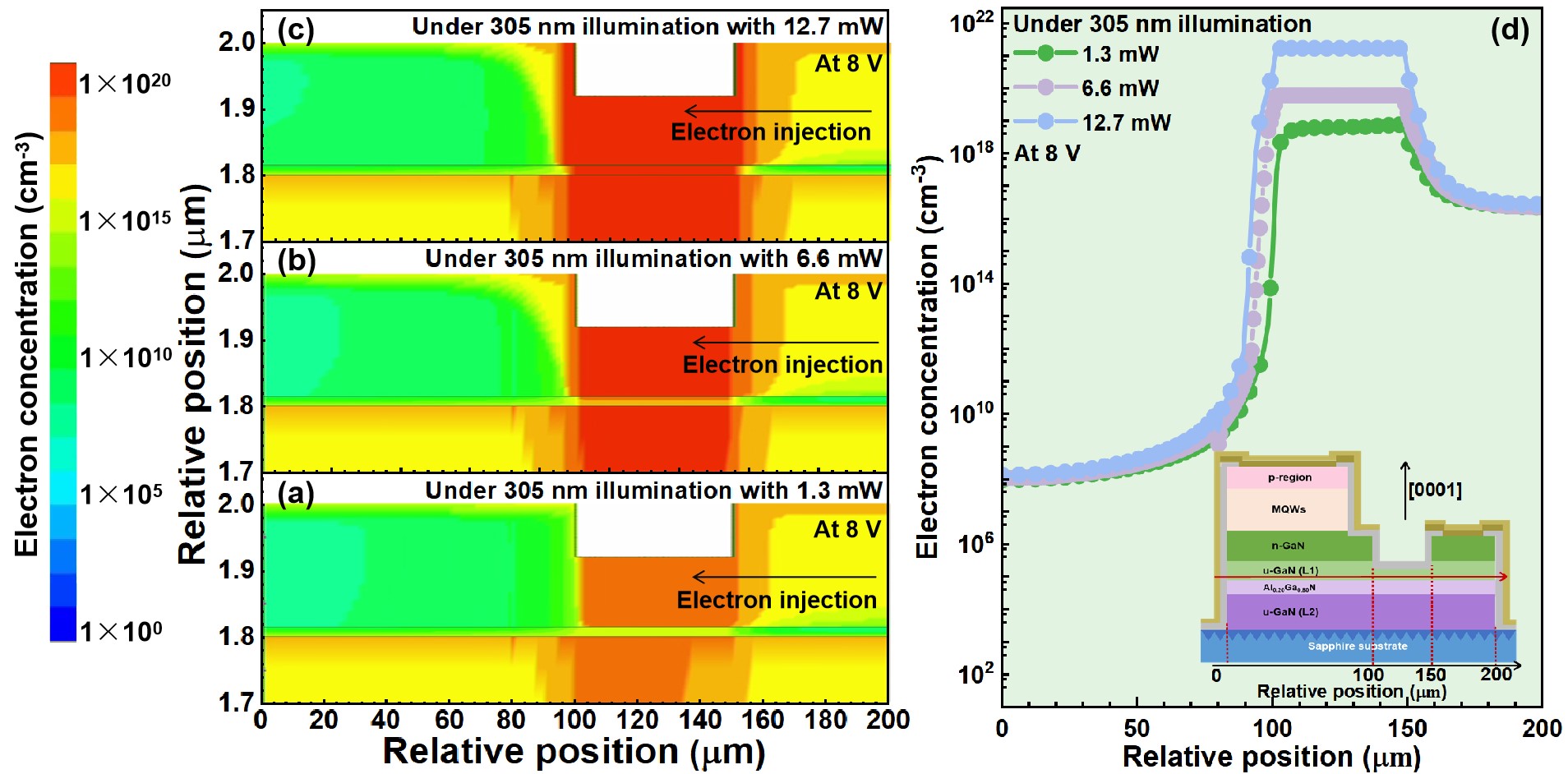
**

**Figure S3.** (a)-(c) Calculated 2-D electron concentration distribution profiles and (d) 1-D concentration distribution profiles for the phototransistor in the integrated optoelectronic device. The data are calculated at the bias of 8.0 V.

Figures S4(a) and S5(a) demonstrate the optical power produced by the mini-LED when the 275 nm and 255 nm DUV excitation light powers are applied, respectively. When the 275 nm DUV excitation source powers are increased to 1.2 mW, 5.6 mW and 10.3 mW, respectively, the optical powers for the mini-LED at the driving voltage of ~10.2 V are increased to 12.7 mW, 15.1 mW and 24.2 mW, respectively. When the 255 nm DUV excitation source powers are increased to 0.7 mW, 3.0 mW and 5.4 mW, respectively, the optical powers for the mini-LED at the driving voltage of ~10.2 V are increased to 6.7 mW, 9.0 mW and 10.1 mW, respectively. The optical power for the mini-LED decreases with the decreasing 275 nm and 255 nm DUV excitation light powers, which indicates that the reduced number for the photo-generated electrons are injected into the MQWs for the mini-LED. Figures S4(b) and S5(b) present the external quantum efficiency (EQE) in terms of the applied bias. The conclusions agree with well with Figs. S4(a) and S5(a). Figures S4(c) and S5(c) show the ratio between the photon numbers for the 460 nm visible light and the 275 nm/255 nm DUV light (N_visible_/N_DUV_) in terms of the injection current. We can find that the number of N_visible_/N_DUV_ increases with the increased injection current. At the same time, a N_visible_/N_DUV_ higher than 20 can be both achieved even when the external DUV light power is low, e.g., 1.2 mW and 0.7 mW in the case for this work. Figures S4(d) and S5(d) present the power-conversion efficiencies (P_visible_/P_UV_) in terms of different external DUV light powers. The power-conversion efficiencies of 13.5 and 14.7 are obtained at the 1.2 mW/275 nm external DUV light and at the 0.7 mW/255 nm external DUV light, respectively. The findings agree with Figs. S4(c) and S5(c), and our fabricated device is able to detect weak DUV light.

**
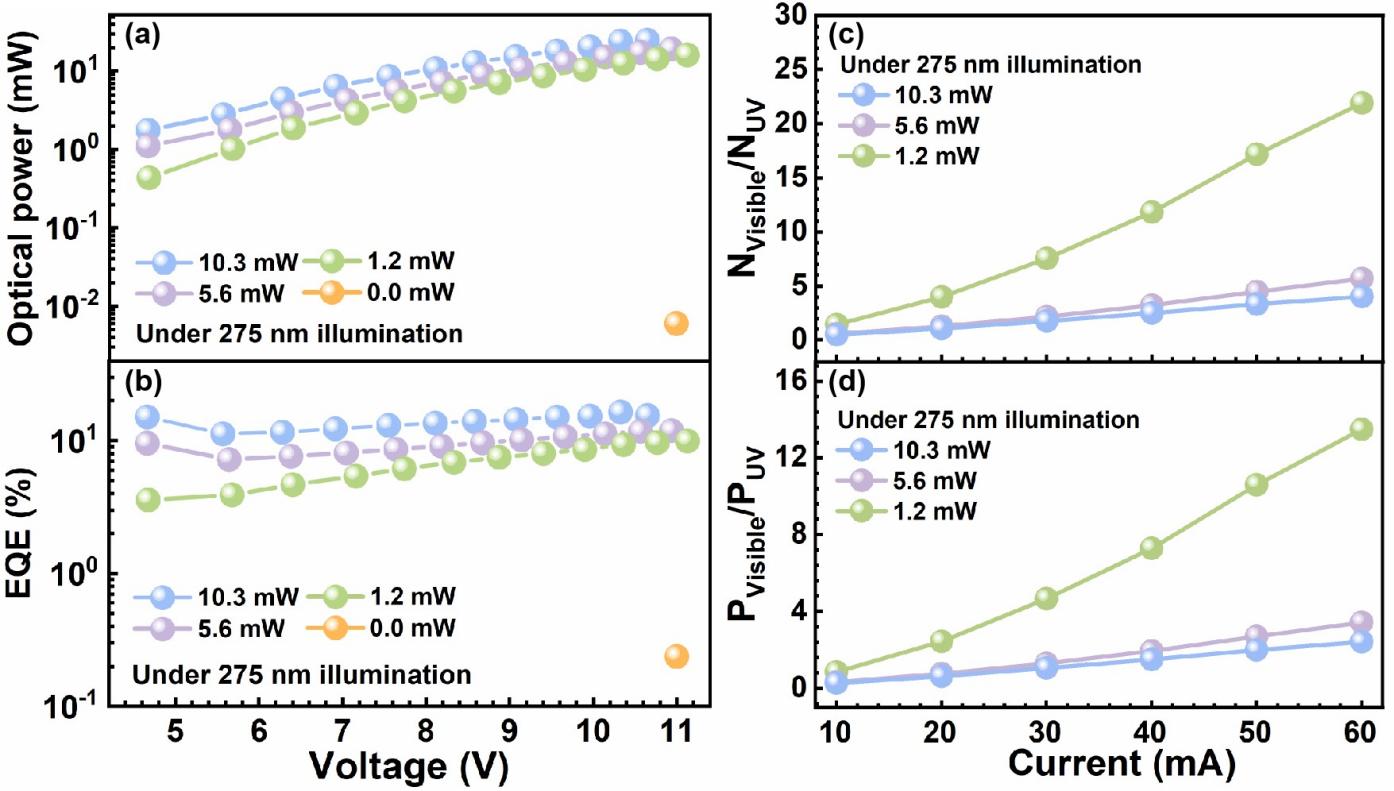
**

**Figure S4.** (a) Optical power and (b) EQE for visible mini-LED in terms of different applied biases when different 275 nm DUV excitation light powers are set. (c) N_visible_/N_UV_ and (d) P_visible_/P_UV_ in terms of the injection current for the integrated optoelectronic device.

**
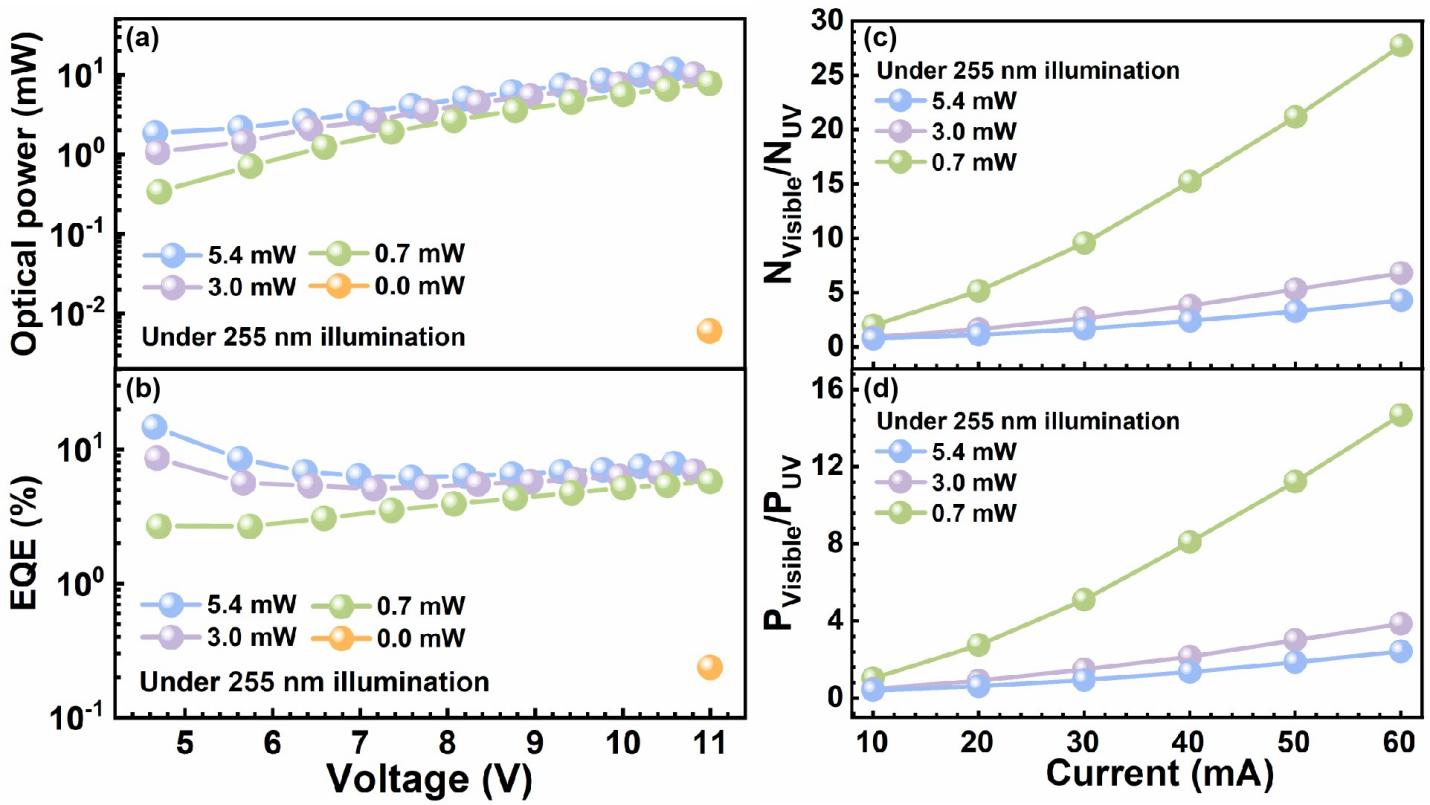
**

**Figure S5.** (a) Optical power and (b) EQE for visible mini-LED in terms of different applied biases when different 255 nm DUV excitation light powers are set, (c) N_visible_/N_UV_ and (d) P_visible_/P_UV_ in terms of the injection current for the integrated optoelectronic device.

**
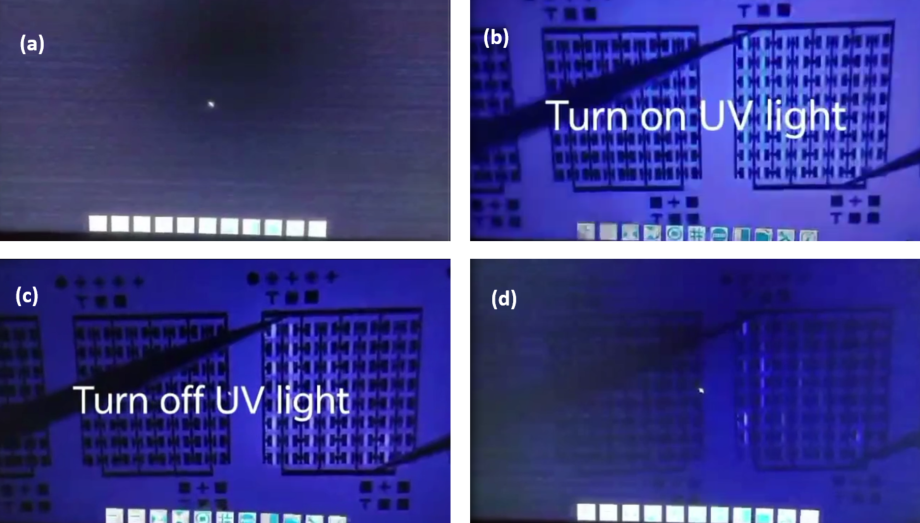
**

**Figure S6.** (a) Image for the blue LED before UV light is turned on, (b) image for the blue LED when UV light is turned on, (c) image for the blue LED when UV light is turned off, and (d) image for the blue LED after UV light is turned off. The video is also presented.

Table S1 compares the response time between our device and other reported devices. In general, different than conventional photodetectors that use reversely biased junction to separate and transport carrier, the device by integrating LED and phototransistor works in forward conduction state. This generates high-level carrier injection and gets carrier scattering involved. Hence, we believe that the response speed for our device can be faster when the device capacitance and deep-level defect density are both decreased.

**Table S1.** Comparison of the response time between our device and other reported devices


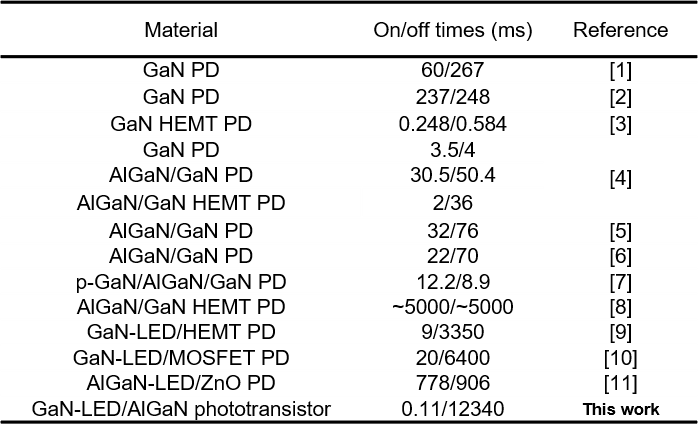


The optical power for the detected UVB and UVC LED chips are measured as shown in Fig. S7(a). We can obtain the optical powers of 1.2 mW, 5.6 mW and 10.3 mW for 275 nm UVC LED at the current levels of 10 mA, 50 mA and 100 mA, respectively. The optical powers for 255 nm UVC LED are 0.7 mW, 3.0 mW and 5.4 mW at the current levels of 10 mA, 50 mA and 100 mA, respectively. The corresponding optical power densities are demonstrated in Fig. S7(b).

**
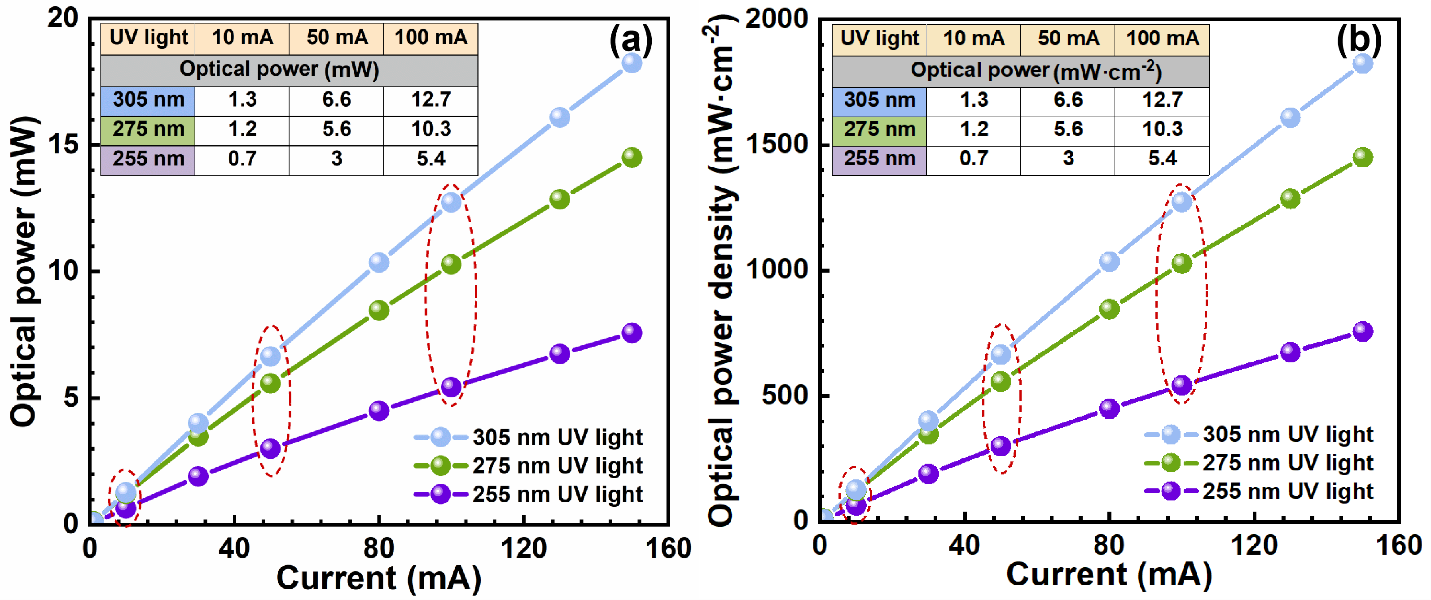
**

**Figure S7.** Optical power in terms of injection current for the UV excitation light source with peak wavelengths of 255 nm, 275 nm and 305 nm, respectively.

According to the description in the main text for this work, the turn-on voltage for the integrated optoelectronic device is ~3.2 V under the 305 nm UV illumination according to Fig. 3(b). Therefore, we present the 1-D and 2-D electron concentration profiles in Fig. S8 when a 3.2 V forward bias is applied. It shows that the electrons concentration in the u-GaN (L1) region is as low as 1.9 ×10^7^ cm^-3^ without the 305 nm UV illumination, which is slightly lower than that at 0 V because of the externally applied opposite electric fields. Thus, without the UV excitation source, the integrated optoelectronic device shows the current even smaller than 1.4 × 10^-4^ mA and the blue emission from the mini-LED is untestable. The electron concentrations in the u-GaN (L1) region are 6.5 × 10^18^ cm^-3^, 6.2 × 10^19^ cm^-3^ and 1.8 × 10^21^ cm^-3^ when the UV excitation light powers are set to 1.3 mW, 6.6 mW, and 12.7 mW, respectively. This ensures the electron current flow and the optical power from the fabricate device.

**
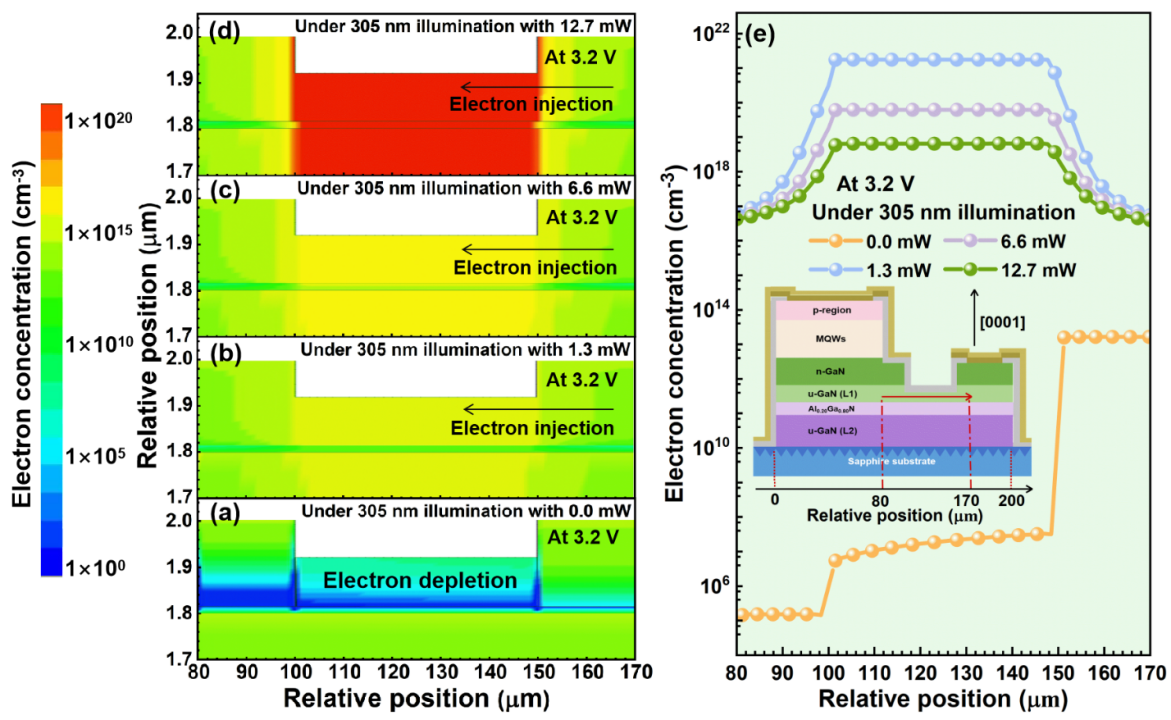
**

**Figure S8.** (a)-(d) Calculated 2-D electron concentration distribution profiles and (e) 1-D concentration distribution profiles for the phototransistor in the integrated optoelectronic device. The data are calculated at the bias of 3.2 V.

The energy bands in the u-GaN (L1)/Al_0.20_Ga_0.80_N/u-GaN (L2) structure at the bias of 3.2 V in different UV illumination conditions are presented in Figs. S9(a)-S9(d), respectively. The energy differences for △E between the conduction band and the quasi-Fermi level for electrons are 0.76 eV, -0.05 eV, -0.28 eV and -2.62 eV when the UV excitation light powers are set to 0.0 mW, 1.3 mW, 6.6 mW, and 12.7 mW, respectively. The △E at 3.2 V is larger than that at 0 V in the u-GaN (L1) layer without the 305 nm UV illumination, indicating that there is still a strong electron depletion region for the integrated optoelectronic device. This is consistent with the electron concentration profiles in Fig. S8(e), which makes the device still in the off-state.

The comparison between Figs. 8(a)/S9(a), 8(b)/S9(b), 8(c)/S9(c) and 8(d)/S9(d) concludes that, when the device is biased, the photon-generated carriers are able to control the on/off-states for the fabricated device.

**
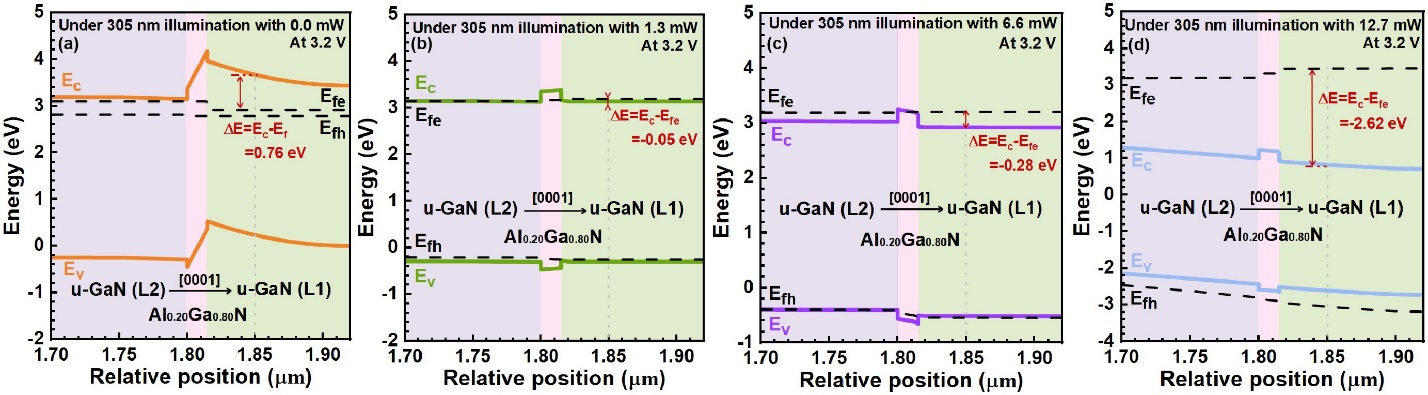
**

**Figure S9.** (a)-(d) Calculated energy band profiles of UV light detection region in the polarization-gated phototransistor for the integrated optoelectronic device at 3.2 V, respectively.

To more clearly show the thickness of the epitaxial layer for GaN-based integrated optoelectronic device, the transmission electron microscopy (TEM) image is shown in Fig. S10(a), which is comprised of 15 nm-thick Al_0.20_Ga_0.80_N layer, 1270 nm-thick u-GaN (L1)/n-GaN layers, 75 nm-thick MQWs and 200 nm-thick p-region. The p-region consists of a 50 nm thick Al-gradient Al_0.15→0.0_Ga_0.85→1.0_N p-type layer and a 150 nm p-GaN layer. As shown in Fig. S10(b), the active region has five pairs of In_0.21_Ga_0.79_N/GaN MQW stacks, for which the thicknesses of the quantum well (QW) and quantum barrier (QB) are 3 nm and 10 nm, respectively.

**
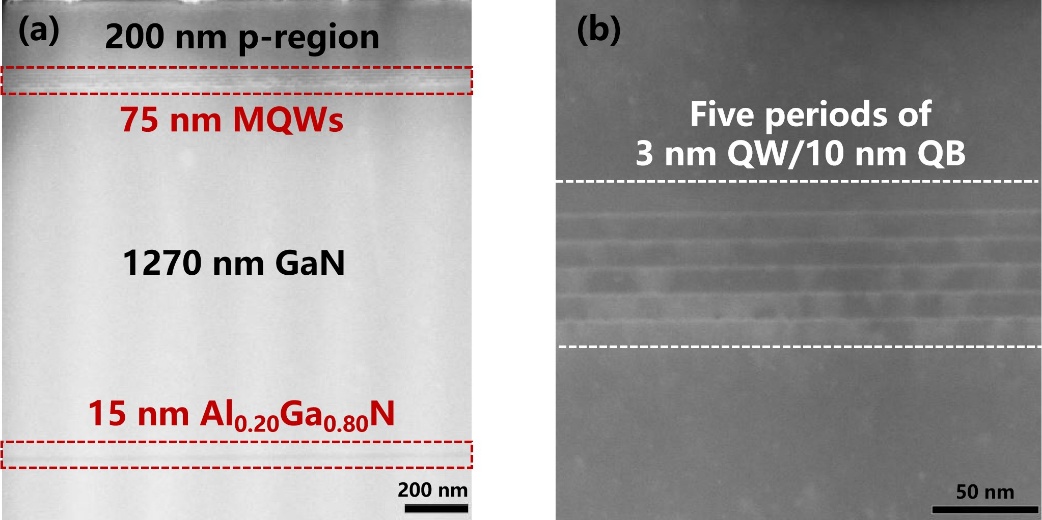
**

**Figure S10.** TEM images of (a) the epitaxial layer from Al_0.20_Ga_0.80_N layer to p-region and (b) MQW region.

**Table S2.** Relationship between the UV light power density and the UV exposure duration time before the skin and eyes are injured

**
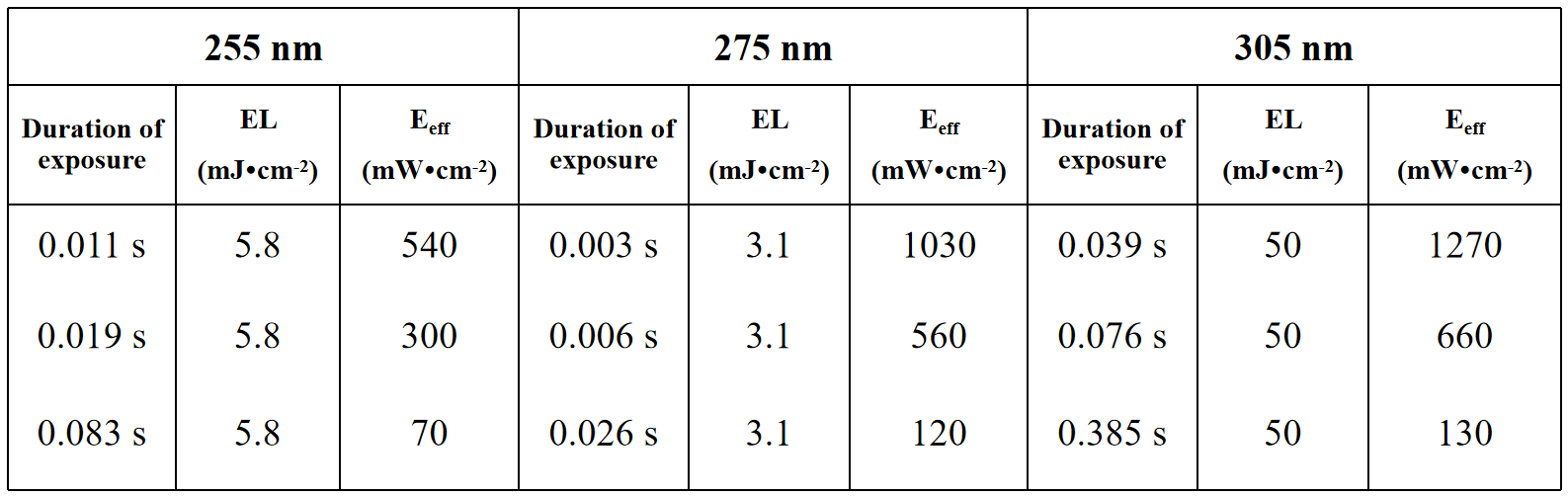
**

With the developed model in Ref. [12], we are able to establish the relationship between the UV light power density and the UV exposure duration time before the skin and eyes are injured. The relationship is presented in Table S1.

1. Prakash, N. et al. Ultrasensitive self-powered large area planar GaN UV-photodetector using reduced graphene oxide electrodes. *Applied Physics Letters* **109**, 242102 (2016).

2. Guo, C. Y. et al. Self-powered ultraviolet MSM photodetectors with high responsivity enabled by a lateral n+/n− homojunction from opposite polarity domains. *Optics Letters* **46,** 3203-3206 (2021).

3. Wang, Y. et al. Light-triggered 2D electron gas in a GaN-based HEMT with sandwiched p-GaN layers. *Optics Letters* **48,** 4376-4379 (2023).

4. Wang, H. D. et al. Ultrahigh-responsivity ultraviolet photodetectors based on AlGaN/GaN double-channel high-electron-mobility transistors. *ACS Photonics* **11,** 180-186 (2024).

5. Satterthwaite, P. F. et al. High responsivity, low dark current ultraviolet photodetectors based on two-dimensional electron gas interdigitated transducers. *ACS Photonics* **5,** 4277-4282 (2018).

6. Pu, Y. H. et al. ITO AlGaN/GaN ultraviolet broadband photodetector with exceeding responsivity beyond the ITO transmittance limitation. *IEEE Electron Device Letters* **45,** 472-475. (2024).

7. Lyu, Q. et al. High gain and high ultraviolet/visible rejection ratio photodetectors using p-GaN/AlGaN/GaN heterostructures grown on Si. *Applied Physics Letters* **117,** 071101 (2020).

8. Wu, S.-C. et al. Nanolayered graphene/hexagonal boron nitride/n-AlGaN heterostructures as solar-blind deep-ultraviolet photodetectors. *ACS Applied Nano Materials* **3,** 7595–7603 (2020).

9. Chen, D. B. et al. GaN-based micro-light-emitting diode driven by a monolithic integrated ultraviolet phototransistor. *IEEE Electron Device Letters* **43,** 80–83 (2022).

10. Piao, J. L. et al. Effective integration of a MOSFET phototransistor to a GaN LED for UV sensing. *Optics Letters* **47,** 3572–3575 (2022).

11. Yu, H. B. et al. Vertically integrated self-monitoring AlGaN-based deep ultraviolet micro-LED array with photodetector via a transparent sapphire substrate toward stable and compact maskless photolithography application. *Laser & Photonics Reviews* 2401220 (2024).

12. International Commission on Non-Ionizing Radiation Protection, Guidelines on limits of exposure to ultraviolet radiation of wavelengths between 180 nm and 400 nm (incoherent optical radiation). *Health Physics*, **87** 171 (2004).
